# Influence of Ensiling Timing and Inoculation on Whole Plant Maize Silage Fermentation and Aerobic Stability (Preliminary Research)

**DOI:** 10.3390/plants13202894

**Published:** 2024-10-16

**Authors:** Jonas Jatkauskas, Vilma Vrotniakiene, Rafael Camargo do Amaral, Kristian Lybek Witt, Bruno leda Cappellozza

**Affiliations:** 1 Department of Animal Nutrition and Feedstuffs, Lithuanian University of Health Sciences, R. Žebenkos 12, 82317 Baisogala, Lithuania; vilma.vrotniakiene@lsmuni.lt; 2 Animal Biosolutions Business Unit, Novonesis, 2800 Kongens Lyngby, Denmark; racl@novonesis.com (R.C.d.A.); kriwi@novonesis.com (K.L.W.); brie@novonesis.com (B.l.C.)

**Keywords:** aerobic exposure, ensiling challenge, *Lentilactobacillus buchneri*, *Lactococcus lactis*, maize silage

## Abstract

Despite efforts to prevent atypical ensiling conditions, such as delayed ensiling or sealing, these issues frequently occur in practice. This study aimed to investigate the effects of delayed ensiling (forage held for 24 h) and sealing, along with inoculation using a blend of *Lentilactobacillus buchneri* and *Lactococcus lactis*, on the characteristics of the resulting silages. Whole-plant maize (*Zea mays* L.) was treated with or without a commercial inoculant and ensiled (36% dry matter) for 60 days in 3.0 L glass containers. The forage was either ensiled immediately or subjected to a 24 h delay before ensiling. During the delay, the forage was either covered or left uncovered. Each treatment was replicated five times. All data were analyzed using the MIXED procedure of SAS statistical software (version 9.4; SAS Institute Inc., Cary, NC, USA). Delaying the ensiling process by 24 h worsens fermentation parameters, significantly increases dry matter (DM) losses (*p* < 0.01), and significantly reduces aerobic stability and the hygienic quality of the silage (*p* < 0.01), as evidenced by higher concentrations of undesirable fermentation products and elevated yeast and mold counts. The inoculation has a significant impact on both forage before ensiling and the characteristics of the resulting silage. Maize forage treated with inoculant showed a lower temperature increase by 8.2–8.1 °C (*p* < 0.01) when delayed for 24 h before ensiling. In silages, it also resulted in a reduced pH (*p* < 0.01); increased concentrations of lactic acid; acetic acid; and 1,2-propanediol (*p* < 0.01); and decreased levels of negative fermentation indicators such as ammonia-N, alcohols, and butyric acid (*p* < 0.01) During both the fermentation and aerobic exposure periods, inoculated silages exhibited up to 36% and 2.6 times lower (*p* < 0.01) dry matter loss, while suppressing the growth of yeasts and molds by up to 2.6 and 3.1 times (*p* < 0.01), respectively, compared to non-inoculated silages. The results of this study support the recommendation to minimize the duration of aerobic exposure of fresh forage during silo filling and to use LAB-based inoculants.

## 1. Introduction

The goal of the initial stages of fermentation is to create an anaerobic (oxygen-free) environment to promote the growth of lactic acid bacteria, which are beneficial for preserving forage [1]. Physical and management factors, such as compaction and sealing, significantly influence the outcome of forage conservation. As an example, prolonged exposure of the ensiled material to the environment can occur due to extended transportation length from the field or delays in filling and sealing the silo. This extended exposure increases the respiration time of plant cells, leading to a decrease in the amount of soluble carbohydrates and potential disturbances in the fermentation processes [2,3]. Ineffective sealing allows air to infiltrate into the silage, leading to aerobic deterioration, nutrient losses, and a decline in feed quality. Air exposure during the ensiling process can delay fermentation by allowing undesired aerobic microbes to metabolize forage components, which slows down the growth of lactic acid bacteria, increases pH levels, and fosters the growth of undesirable organisms such as yeasts and molds. This negatively impacts the nutritional profile of the silage and increases the risk of spoilage, especially during feed-out [2,4,5,6]. Achieving rapid anaerobiosis, or the exclusion of air, is essential to effective fermentation. Under anaerobic conditions, lactic acid bacteria thrive, producing lactic acid that lowers the pH and preserves the silage [2,7,8]. Additives, particularly inoculants containing lactic acid bacteria like *Lactobacillus buchneri*, are crucial for enhancing fermentation, controlling undesirable microbial activity, and improving the overall preservation and nutritive value of the silage [4,9]. The presence of aerobic microorganisms increases pH and generates heat, leading to nutrient degradation and a reduction in silage quality [10]. The use of additives is essential in controlling the fermentation process and enhancing aerobic stability, which is vital for preserving silage quality [11].

Previous studies have primarily focused on understanding the changes occurring during the initial aerobic phase of fermentation, with less emphasis on the impact of atypical ensiling conditions, such as interrupted ensiling, on silage fermentation and aerobic stability. This gap in the literature highlights the need for further research into how these conditions affect silage characteristics. Conducting experiments using field or lab-scale silos could provide valuable insights into optimizing silage preservation techniques and improving aerobic stability in silage systems. Based on this rationale, we hypothesized that interrupted ensiling would negatively affect the characteristics of whole-plant maize silage, but that adding a LAB-based inoculant could mitigate these adverse effects. Consequently, this study aimed to investigate the effects of interrupted overnight ensiling and the application of a LAB-based inoculant on the fermentation characteristics, dry matter recovery, and aerobic stability of whole-plant maize silage.

## 2. Results

### 2.1. Characterization of Maize Forage after Test Conditions before Ensiling in Mini-Silo

#### 2.1.1. Forage Temperature and Contamination by Yeast and Mold

A significant temperature increase (*p* < 0.01) was observed in both non-inoculated and inoculant-treated maize forages after being stored in boxes for 24 h before ensiling into a mini-silo (Table 1). The non-inoculated forages (T1C–T2C) showed a significantly higher temperature increase by 8.2–8.1 °C (*p* < 0.01) compared to the inoculant-treated forages (T4I–T5I). There were differences in the time it took for the temperature to increase by 3 °C up to the ambient conditions. The non-inoculated forages (T1C–T2C) reached a temperature 3 °C higher than ambient 6.0 to 8.4 h faster (*p* < 0.01) than the inoculant-treated forages (T4I–T5I). The simulation of overnight covered or uncovered forages did not show significant differences in yeast and mold counts. However, forage treated with inoculant had significantly lower yeast and mold counts (*p* < 0.01) and, as expected, a significantly higher number of lactic acid bacteria (LAB) (*p* < 0.01) compared to the untreated forage.

#### 2.1.2. Forage Chemical Composition

The timing of ensiling and the use of inoculation did not significantly affect the DM content or nutrient composition of maize forage prior to ensiling (Table 2). Whether the maize forage was ensiled immediately or held for 24 h, with or without inoculation, there were no noticeable differences in DM content or nutrient composition

### 2.2. Characterisation of Maize Silage Ensiled into Mini-Silo and Fermented for 60 Days

#### 2.2.1. Nutrient Composition and Microbial Characteristics of Maize Silage Fermented for 60 Days

The data in Table 3 indicate that inoculated silage, whether promptly ensiled (T3I) or ensiled after a 24 h delay, either uncovered (T4I) or covered (T5I), had a 2.1–2.5% higher (*p* < 0.01) DMc content compared to the non-inoculated silage (T0C–T2C). All inoculated silages (T3I–T5I) demonstrated a 34.1–36.2% reduction (*p* < 0.01) in dry matter (DM) loss compared to non-inoculated silages, regardless of the timing of ensiling or covering conditions. Silage that was not inoculated and was ensiled after 24 h, either uncovered (T1C) or covered (T2C), showed a significantly lower (*p* < 0.01) crude protein concentration compared to the other treatments (T0C and T3I–T5I). The non-inoculated silages, ensiled after 24 h either uncovered (T1C) or covered (T2C), contained significantly lower (*p* < 0.01) starch content compared to the other silages (T0C and T3I–T5I).

As expected, the LAB count in inoculant-treated silages (T3I–T5I) was found to be 1.1–1.4 log_10_ cfu g^−1^ higher (*p* < 0.01) than in non-inoculated silages (T0C–T2C). Concurrently, inoculated silages (T3I–T5I) showed a 1.0–1.2 log_10_ cfu g^−1^ lower (*p* < 0.01) yeast count and a 1.2–1.9 log_10_ cfu g^−1^ lower (*p* < 0.01) mold count compared to non-inoculated silages (T0C–T2C). Among the inoculated silages, the lowest yeast count was found in the promptly ensiled (T3I) silage, while the promptly ensiled (T0C) silage had the lowest yeast and mold counts among the non-inoculated silages (Table 3).

#### 2.2.2. Fermentation Characteristics of Maize SILAGE Fermented for 60 Days

Table 4 presents the fermentation characteristics of maize silage ensiled in mini-silos under various treatments, including promptly ensiled or ensiled after a 24 h delay, with or without inoculant treatment, and fermented for 60 days. The data indicate significant differences in fermentation characteristics among the treatments, particularly in terms of pH, lactic acid, acetic acid, and ethanol. For instance, the treatments with inoculant, whether ensiled promptly or after a 24 h interruption uncovered and covered (T3I, T4I, and T5I), generally exhibited pH values that were 0.08, 0.16, and 0.10 units lower (*p* < 0.01), respectively, compared to the non-inoculated treatments (T0C, T1C, and T2C). The highest pH value (3.98; *p* < 0.01) was observed in the silages ensiled with a 24 h interruption and left uncovered (T1C). The use of inoculant results in a significant reduction (*p* < 0.01) in NH_3_-N, alcohols and butyric acid content compared to non-inoculated treatments. Notably, when inoculant was used and ensiling was performed either promptly (T3I) or with a 24 h delay (T5I), the ammonia-N content was the lowest (*p* < 0.01) among all groups, ranging between 38.03 and 38.56 g/kg total N. Alcohol and ethanol formation was the highest (34.32 and 30.87 g kg^−1^ DMc, *p* < 0.01) in T1C silages among all the treatments. It should be noted that the use of the inoculant reduced butyric acid formation by an order of magnitude. Furthermore, a significant increase (*p* < 0.01) in lactic acid, acetic acid, and 1,2-propanediol content was observed in corn silages where inoculant was applied, either in promptly ensiled (T3I) or in silages ensiled with a 24 h delay (T4I, T5I), compared to non-inoculated silages (T0C–T2C). In particular, the highest increase in lactic acid content (58.7%, *p* < 0.01) and 1,2-propanediol content (4.5 times, *p* < 0.01) was observed in the T3I silage, while the highest increase in acetic acid content (48.7%, *p* < 0.01) was found in the T4I silage.

### 2.3. Characteristics of Maize Silage Aerobic Stability

A comprehensive evaluation of the aerobic stability of silage involved examining various factors to assess how well the silage withstands exposure to air. This evaluation included analyzing chemical composition, fermentation parameters, pH value, nutrient composition alterations, weight loss, and yeast and mold counts at day 7 of aerobic exposure. Additionally, temperature changes during the 17-day aerobic exposure period and microbial activity, such as yeast and mold counts on the end of aerobic stability test, provided a holistic view of the silage deterioration under aerobic conditions (Table 5, Table 6 and Table 7).

After 7 days of exposure to air, the inoculated and promptly ensiled (T3I) maize silage conserved 8.1% more (*p* < 0.01) dry matter and 9.2% more (*p* < 0.01) starch compared to the non-inoculated treatment (T1C), which was ensiled with a 24 h delay and left uncovered (Table 5). All inoculated silages (T3I–T5I) showed a significant reduction (*p* < 0.01) in dry matter (DM) loss, ranging from 58.7% to 2.4 times less, compared to non-inoculated silages (T0C–T2C), depending on the timing of ensiling and covering conditions. Among these, the inoculated and promptly ensiled maize silage (T3I) exhibited the lowest (*p* < 0.01) DM loss. Inoculant-treated (T3I–T5I) silages preserved 2.6–3.0% higher (*p* < 0.01) concentrations of metabolizable energy compared to non-inoculated (T0C–T2C) silages.

The results of fermentation variables after 7 days of aerobic exposure (Table 6) clearly demonstrated differences compared to the fermentation product characteristics observed at the silo opening on day 60 of fermentation (Table 4). Aerobic exposure for 7 days led to decreased concentrations of fermentation products and an increased pH. Unfortunately, this data was not analyzed statistically. On day 7 of aerobic exposure, the inoculated silages (T3I–T5I) showed significantly lower (*p* < 0.01) pH values by 1.31, 1.12, and 1.18 units, respectively, compared to the non-inoculated silages (T0C–T2C), depending on the initial ensiling time and covering conditions. The T3I silages showed the lowest (3.9, *p* < 0.01) pH value among all treatments. The lowest ethanol concentration (2.64 g kg^−1^ DMc, *p* < 0.01) was detected in the T3I silages, while the highest (8.30 g kg^−1^ DMc, *p* < 0.01) was observed in the T2C silages. The inoculated silages (T3I–T5I) exhibited significantly lower (*p* < 0.01) butyric acid levels—reduced by 3.5 times, 34%, and 3.5 times, respectively—compared to the non-inoculated silages (T0C–T2C), depending on the initial ensiling time and covering conditions. The highest concentration of ammonia-N (37.55 g/kg total N, *p* < 0.01) was found in the T2C silages among all treatments. Aerobic exposure for 7 days reduced the lactic acid concentration of the inoculant-treated silages (T3I–T5I) to 42.19, 24.25, and 32.11 g kg^−1^ DMc, respectively, while the lactic acid concentration of the non-inoculated silages (T0C–T2C) was 10.18, 6.44, and 9.48 g kg^−1^ DMc, respectively. The differences between the inoculant-treated and non-inoculated silages were significant (*p* < 0.01). The non-inoculated silages (T0C–T2C) exhibited significantly lower (*p* < 0.01) acetic acid and 1,2-propanediol levels—reduced by 2.5, 3.1, and 2.1 times, respectively, for acetic acid, and by 7.6, 3.8, and 4.3 times for 1,2-propanediol, respectively, compared to the inoculated silages (T3I–T5I), depending on the initial ensiling time and covering conditions. Inoculated silages (T3I–T5I) maintained aerobic stability for 250.8, 165.6, and 192.0 h, respectively—significantly longer (*p* < 0.01) and reached peak temperatures 4.6 °C, 3.02 °C, and 3.9 °C lower (*p* < 0.01), respectively, when exposed to air, compared to non-inoculated silages (T0C–T2C), depending on the initial ensiling time and covering conditions (Table 7). By the end of 7 and 17 days of aerobic exposure, inoculated silages (T3I–T5I) exhibited 3.1–2.9 times and 1.8 times, respectively, lower (*p* < 0.01) yeast counts and 3.3–2.9 times and 1.7–2.9 times, respectively, lower (*p* < 0.01) mold counts compared to non-inoculated silages (T0C–T2C), regardless of the initial ensiling time and covering conditions.

## 3. Discussion

Farm-scale silos often face delays in being sealed and fully hermitized, whether left uncovered or covered, leading to exposure to air for varying periods, which can have negative consequences to the quality of the ensiled material, leading to decreased feed intake up to 53% as indicated by Brüning et al. [2] and increased DM losses of up to 11%, as described by Borreani et al. [12]. Indeed, the respiration of aerobic microbes generates heat, which increases the temperature of the silage over 40 °C compared to the ideal basal temperature (15–25 °C); subsequently increases the risk of spoilage, leading to higher wastage rates. Spoiled silage not only reduces the available feed to the producer but can also pose health risks to animals when consumed [3] because it promotes the proliferation of non-ideal bacteria, such as the *Clostridium* genus. In the present study, various conditions simulating real-life farming practices were assessed to determine their impact on the ensiling process of whole-plant maize forage. The simulation included different scenarios where ensiling was either performed promptly or interrupted overnight. During these interruptions, the maize forage was either covered with a plastic film or left uncovered. Additionally, the forage was either inoculated with silage additive (150,000 cfu g^−1^ of fresh crop) or left uninoculated. This experiment was conducted on a laboratory scale to closely monitor and analyses the outcomes.

### 3.1. Characterisation of Maize Forage after Test Conditions before Ensiling in Mini-Silo

A significant temperature increase (*p* < 0.01) was observed in maize forage, both inoculated and non-inoculated, after being stored in boxes for 24 h prior to ensiling into a mini-silo. The heating is a sign of aerobic respiration, and that the silage material may be compromised. Slow silo filling and delayed silo sealing can lead to an initial growth of spoilage organisms and a reduction in the production of lactic acid, which is crucial for lowering pH and stabilizing the silage [13]. The study by Pauly et al. [14] demonstrated that a 24 h delay in sealing resulted in a forage temperature increase to 37 °C, suggesting that oxygen exposure caused substantial heating. This delay also led to a pH rise from 5.80 to 6.73, indicating a reduction in forage quality. According to Kim and Adesogan [15], aerobic exposures longer than 10 h, as frequently can be observed at the farm level may impair the following fermentation process by reducing soluble carbohydrate content. Additionally, prolonged aerobic exposure may lead to greater contamination by yeasts [6]. However, the forage treated with inoculant exhibited temperatures that were 8.2 °C lower (*p* < 0.01) and had 15–17% less (*p* < 0.01) yeast and 26–28% (*p* < 0.01) less mold contamination compared to the non-inoculated forage. These findings suggest that the inoculating and covering the forage can help mitigate the temperature increases that may potentially indicate better preservation of the silage by controlling the aerobic heating. The timing and use of inoculation did not have a significant impact on the dry matter content and nutrient composition of the maize forage before ensiling. Both uninoculated and inoculated whole-plant maize, whether fresh or exposed to air for 24 h (covered or uncovered), had similar chemical compositions. The simulation of overnight uncovered/covered forage did not show significant differences in mold count, but 24 h of air exposure before ensiling led to a significant increase in yeast counts in uninoculated silages. Moreover, yeast counts were significantly lower in inoculated silages compared to uninoculated ones with a 24 h delay and no significant differences in yeast counts were observed between overnight covered and uncovered silages. This result is particularly interesting as it supports the concept that *Lactococcus lactis* could have the ability to scavenge oxygen. This suggests possible improved fermentation of the silage. In our trial, the primary benefit of these treatments lies in improving the preservation conditions rather than altering the nutritional quality of the forage.

### 3.2. Chemical (Nutrient) Composition, Fermentation Profile and Microbial Parameters of Maize Silages after 60 Days of Fermentation

Overall, all silages were well-fermented with no signs of spoilage and exhibited good sensory properties at the time of silo opening, indicating successful ensiling processes. Silages showed minor differences in chemical composition and most fermentation variables, which were influenced by the forage conditions before ensiling. However, inoculated silages consistently exhibited higher (2.1–2.5 percentage units) dry matter corrected for the volatiles content compared to uninoculated silages, regardless of the forage conditions before ensiling. The positive effects of the LAB inoculant on DMc content can be attributed to its dominance during fermentation, resulting in a more efficient fermentation process, as reflected by the rapid achievement of a low pH (3.80–3.82) and composition of fermentation variables. Silage that was not inoculated and ensiled after 24 h delay, uncovered or covered, showed lower (3.0–5.7 percentage units) crude protein content when compared with promptly ensiled and inoculated silages. The changes observed in the present study can be attributed to the process of carbohydrate oxidation that releases heat and increases NDIP and ADIP contents through the Maillard reaction, which can reduce protein availability [16]. Moreover, the reduction in CP content is attributed to prolonged exposure to air, which enhances proteolysis [17,18,19,20,21]. Our data support findings of proteolytic activity by *Lactobacillus buchneri* and consistently lower ammonia-N concentrations when *Lactobacillus buchneri* is combined with homofermentative LAB [22]. 

The ammonia-N concentration in the silage reflects the rate of protein degradation during ensiling [23]. Taylor et al. [24] also reported a reduction in ammonia-N concentration (from 0.18% to 0.16%, *p* < 0.05) in whole-plant barley silage inoculated with *Lentilactobacillus buchneri*.

Our results demonstrate that air entry during a 24 h period before ensiling significantly increased yeast and mold counts in the silages up to 3.30–3.18 log10 cfu g^−1^, respectively. Weiss et al. [5] indicated that factors such as increased availability of WSC, air ingress and higher temperature during fermentation promote yeast growth in silage up to 7.27 log10 cfu g^−1^. In our study, inoculant treatment was effective in reducing yeast and mold counts in the silages up to 1.40–1.00 cfu g^−1^, respectively, while simultaneously increasing the number of LAB up to 7.56–7.92 cfu g^−1^, regardless of the initial conditions of the maize forage before ensiling. Driehuis et al. [25] have already described these effects when additives composed of *Lactobacillus buchneri* alone or in combination with homofermentative LAB reduced (at least *p* < 0.05) mold and yeast counts, increased silage fermentation quality, and greatly improved aerobic stability. Ethanol can be produced by various microorganisms, primarily yeasts, and is typically present at low concentrations in silages (0.5–1.5%) [26]. Higher ethanol concentration (2.6–3.1%) in the uninoculated silage that was delayed in ensiling for 24 h and left uncovered correlated with the highest yeast counts observed in this experiment, confirming that yeast species require a certain oxygen level for anaerobic alcohol production [23,27]. Furthermore, forage that was uninoculated with a 24 h ensiling delay and uncovered had higher ethanol and ammonia-N concentrations and lower levels of 1,2-propanediol compared to the treatment that was promptly ensiled. These findings could be attributed to the aerobic activity of enterobacteria or to oxygen-induced changes of the metabolism by LAB [5].

Moreover, all inoculated silages presented a significantly lower pH than uninoculated ones (3.80–3.82 vs. 3.88–3.98) due to a higher organic acid concentration such as lactic and acetic acids, and the highest pH value (3.98) was observed for the silages ensiled with pause of 24 h uncovered. In general, LAB compete with undesirable aerobic microbes limiting the occurrence of aerobic microbes and exhibiting a lower bacterial diversity compared to natural silages and when their development prevails it results in a drop in pH [1,28]. Some studies have reported that the use of viable LAB bacteria additives did not always increase the number of LAB in silage [29]. In our study, an increase in LAB numbers was observed in all inoculated silages despite timing of ensiling, and this is likely to be related to the observed increase in organic acids and the resulting decrease in pH value compared to the uninoculated ones [1]. The change in fermentation end products affects the fermentation process and dry matter (DM) losses. In silages that have been inoculated, these changes lead to significantly lower fermentation or DM losses (4.0–4.4% inoculated vs. 6.2–7.0% for non-inoculated), regardless of the timing of ensiling. The DM losses in our study were correlated with high yeast number, alcohol, and ammonia-N formation in uninoculated forage that was delayed in ensiling for 24 h and left uncovered. The used inoculant additive in our study were successful in reducing DM losses to 4.4% or lower, which is very close to the 5.2% value suggested by Borreani et al. [12].

According to Rooke and Hatfield [30] and McDonald et al. [18], DM losses are generally much higher when bacteria other than lactic acid bacteria play a dominant role in fermentation. The present results confirmed the importance to stop as soon as possible air inclusion to prevent intensive aerobic conversion processes during the first days of storage. Bruning et al. [2] found in the experiments with maize that a delay of sealing by four days resulted in fermentation losses of up to 11%. Similarly, Nutcher et al. [31] reported that delaying the sealing of maize silage by one day led to a 27.2% increase in organic matter (OM) losses in the top 45 cm of the silage, compared to immediate sealing (156 g kg^−1^ vs. 123 g kg^−1^). This data clearly showed that any undesired fermentation processes that generate CO2 must be suppressed to maintain the highest DM recovery possible and must be restricted development of microorganisms that usually grow under conditions unfavorable for the development of the desired microflora [32]. Therefore, inoculant treatment significantly influenced the fermentation process of maize silage. It effectively reduced the pH, regardless of air exposure, and maintained higher concentrations of beneficial fermentation acids initially. Additionally, inoculant treatment enhanced the production of 1,2-propanediol, a compound associated with favorable fermentation outcomes. Conversely, uninoculated silages, particularly those subjected to 24 h pauses and left uncovered before ensiling, exhibited higher levels of undesirable fermentation products such as ethanol, ammonia-N, and butyric acid. This indicates that inoculants play a crucial role in improving the quality and reducing aerobic deterioration of maize silage by promoting beneficial fermentation pathways and minimizing the formation of harmful products.

### 3.3. Characterization of Aerobic Stability

Evaluating temperature dynamics, pH value, weight loss, yeast, and mold counts are all important parameters for determining how well silage holds up when exposed to air after the fermentation process. Monitoring temperature changes can indicate microbial activity and heat generation, while pH value can reflect the acidity level, which affects microbial growth. Weight loss measures the extent of dry matter loss due to aerobic deterioration, and yeast and mold counts indicate the presence of spoilage organisms. This comprehensive approach provides valuable insights into the aerobic stability and quality of the silage [5,28,31]. Our findings suggest that the inoculant composed of *Lentilactobacillus buchneri* and *Lactococcus lactis* consistently reduced silage deterioration during the aerobic exposure period and improved aerobic stability compared to non-inoculated silage. This was attributed to lower yeast counts, as well as higher concentrations of acetic acid and 1,2-propanediol. Inoculated silages, regardless challenged ensiling conditions of maize forage before ensiling, maintained aerobic stability for a longer period when exposed to air and exhibited lower counts of yeasts 1.8–3.3 times and mold 1.7–2.9 times than uninoculated silages. The data from the present study show a positive correlation between the concentration of acetic acid, 1,2-propanediol, lower yeast counts, and increased aerobic stability. This relationship between yeast counts, acetate level, 1,2-propanediol and aerobic stability has been described by other authors [5,33]. Uriarte-Archundia et al. [34] reported increased up to 7.65 log10 cfu g^−1^ lactate-assimilating yeasts and reduced aerobic stability when maize forage sealing was delayed by two days compared to immediate sealing. At day 7 of the aerobic stability test, despite different forage conditions before ensiling, inoculated silages had the significantly lower pH value (3.90–4.10 vs. 5.22–5.36) and DM loss ranging from 58.7% to 2.4 times, as well as yeast and mold counts, compared to the uninoculated ones. The increase in pH during exposure to air is an important indicator of silage deterioration and can be attributed to the loss of lactic acid [22], which was very clear in the uninoculated silages in the present study. Aerobic fermentation occurs in the silage after opening the silo, carried out by microorganisms that utilize organic acids such as lactic acid, soluble sugars, and ethanol as main substrates. When microorganisms, primarily yeasts, are exposed to air, they consume this lactic acid, causing an increase in pH [5,35]. Driehuis et al. [25] showed that the addition of *Lentilactobacillus buchneri* reduced the survival of yeasts during the anaerobic of silage fermentation phase from 4.5 to <2.0 log10 cfu g^−1^ and inhibited yeast growth during exposure of silage to air from 3.8 to <2.0 log10 cfu g^−1^.Tabacco et al. [36] and Auerbach and Nadeau [22] indicated that *Lentilactobacillus buchneri* can inhibit aerobic deterioration of silage by fermenting lactic acid to acetic acid and limiting the growth of yeasts and clostridia. Therefore, the application of the inoculant (*Lentilactobacillus buchneri* and *Lactococcus lactis*) impacted maize forage both prior to ensiling and over the long term, improving fermentation characteristics and reducing dry matter DM losses in the silage, enhanced aerobic stability and minimized DM loss during the aerobic exposure phase.

## 4. Materials and Methods

### 4.1. Study Site and Experimental Design

This study was conducted at the Experimental Farm of the Institute of Animal Science of Lithuanian University of Health Sciences, situated in the mid-region of Lithuania. The soil used for cultivation was simple carbonate leach soil. The average early maturing (FAO number 200/220) forage maize hybrid used was KWS STEFANO, developed by KWS SAAT SE & Co. KGaA, Lochow, Germany. The maize planting took place on 6 May 2019 at a density ranging from 80,000 to 90,000 seeds per hectare. Prior to sowing, 400–500 kg/ha of complex fertilizers N210 P80 K120 were applied, and after germination, a total of 90 kg of N per hectare was provided. The whole-plant maize was harvested at the late dough stage of grain on 18 September 2019 using a Claas Jaguar 840 forage harvester from Harsewinkel, Germany. The harvester was set to chop the maize to a theoretical length of 10 mm.

After harvesting, the maize forage was transported to a laboratory for silage preparation. In the laboratory, the chopped maize forage was divided into two equal portions (200 kg each), thoroughly mixed separately, and samples were taken to determine the chemical composition and the counts of yeast and mold. One portion of the forage remained untreated, while the other portion was treated with an inoculant containing LAB strains in a 50:50 ratio of *Lentilactobacillus buchneri* DSM 22,501 and *Lactococcus lactis* DSM 11,037 (Novonesis, Hørsholm, Denmark). The inoculant was applied at a rate of 1.5 × 10^5^ colony-forming units (cfu) per gram of fresh crop. After dividing the maize and applying the inoculant, the chopped forage was weighed again to ensure the correct application rate of the inoculant. Samples of water and inoculant suspension were collected and immediately analyzed for LAB counting using the ISO method 15,214 [37]. This rigorous procedure ensured that the maize forage was properly inoculated before proceeding with further experimentation on silage fermentation characteristics, dry matter recovery, and aerobic stability. Then, the untreated and inoculant-treated chopped maize was processed as follows: a portion of each treatment was promptly ensiled into 3 L mini-silos. Another portion (30 kg per treatment) was placed into 80 L plastic boxes (70 cm length × 40 cm width × 28 cm height). The fresh maize material was pressed by foot to fill 2/3 (60 L) of the box, achieving a density of 174 ± 3 kg/m^3^. These boxes were then left for 24 h, either uncovered or covered.

This technique simulated potential farm conditions where the ensiling process was interrupted overnight, with or without a plastic film covering the ensiled forage. The forages, left either uncovered or covered for 24 h, were sampled before being placed into mini-silo to determine their chemical composition, as well as yeast and mold counts. The experiment followed a completely randomized design with six treatments and 5 replicates per treatment as described below.

T0C: Non-inoculated, promptly ensiled.
T1C: Non-inoculated, with a 24 h ensiling pause, uncovered.
T2C: Non-inoculated, with a 24 h ensiling pause, covered.
T3I: Inoculated, promptly ensiled.
T4I: Inoculated, with a 24 h ensiling pause, uncovered.
T5I: Inoculated, with a 24 h ensiling pause, covered.

### 4.2. Ensiling into Mini-Silo

The mixed forage was weighed for each mini-silo to ensure the correct amount of forage was used to achieve the target density of 200 kg DM/m^3^. The weighed forage was then placed into 3-L glass jars by hand. During the filling process, periodic tamping was carefully performed to minimize the air headspace within the jars. Each mini-silo (glass jar) was weighed when empty and then again after filling to determine the actual amount of forage ensiled. Each jar was labeled individually for identification. The jars were closed and made airtight with caps within 30 min of filling. The sealed jars were stored at room temperature (20 °C) in the dark for 60 days. This method ensured that each mini-silo was prepared uniformly, providing a controlled environment for studying the ensiling process.

### 4.3. Mini-Silages Sampling, Chemical and Microbiological Analyses

Sixty days post-ensiling, the 3 L glass jars (mini-silos) were opened, and each mini-silo was weighed to determine the fresh weight loss since the beginning of the fermentation period. The initial weight (before fermentation) and the final weight (after 60 days) was used to calculate fresh weight loss. The initial and final weights and the dry matter content of the material were used to determine the DM loss during the fermentation period. Representative samples from each mini-silo were taken for further chemical and microbial analysis. These analyses included measurements of dry matter (DM pH; crude protein; neutral detergent fiber (NDF); acid detergent fiber (ADF); water-soluble carbohydrates (WSC); starch, metabolizable energy (ME) (calculated); volatile fatty acids (VFA—includes key fermentation products such as lactate, acetate, butyrate, and propionate); ammonia-N; yeast; and mold; as well as total LAB counts. Chemical and microbial analyses were conducted following the procedures described by Jatkauskas et al. [38].

### 4.4. Aerobic Stability Evaluation of the Silages

The aerobic stability of silages was assessed through a comprehensive evaluation of several parameters during a 17-day aerobic exposure period. The temperature dynamics of the aerobically exposed silages were continuously recorded throughout this period, following the methodology described by Jatkauskas et al. [38]. Yeast and mold counts were measured on the 7th day of aerobic exposure to determine microbial activity and potential spoilage. Final yeast and mold counts were taken at the end of the 17-day period to assess the overall microbiological stability of the silages. The pH levels of the silages, changes in the chemical composition, key fermentation variables, and the loss of fresh weight were monitored during the 7-day aerobic exposure period to assess the nutrient preservation and any degradation occurring during aerobic exposure.

### 4.5. Calculations for Corrected Dry Matter (DMc) Concentration, Dry Matter Loss and Metabolizable Energy (ME)

To correct for the loss of volatile fermentation products during the drying process of maize silage samples, the equation proposed by Weissbach and Strubelt, [39] was used.

(DMc = DMn + 0.95 FA+ 0.08 LA + 0.77 PD +1.00 OA (g kg^−^^1^ FM),

where FA = fatty acids (C2 . . . C6), LA = lactic acid, PD = 1,2 propanediol, and OA = other alcohols C2 . . . C4, including 2,3-butanediol. Each component (FA, LA, PD, OA) was measured in grams per kilogram of fresh weight and added to the not corrected dry matter (DMn) according to the specified coefficients.

The DM loss was calculated based on the initial DM content at ensiling and the DMc content of the silage. Considering the DM content of the forage and silage, the DM loss was calculated using the following equation:

DM loss = (DM at ensiling − DMc silage)/DM at ensiling.

The metabolizable energy (ME) was calculated using the following equation:

ME (MJ/kg DM) = 14.03 − 0.01386 × Crude fiber − 0.01018 × Crude ash,

where: CP—crude protein, g/kg DM, EE—ether extracts, g/kg DM CFiber—crude Fiber, g/kg DM, NfE—nitrogen free extracts, g/kg DM [40].

### 4.6. Statistical Analysis

All data were analyzed with MIXED procedure of the SAS Statistical Software (version 9.4; SAS Inst. Inc., Cary, NC, USA). The experiment was analyzed as a complete randomized design with 6 treatments and five replicates per treatment. The mini silo/jar was used as the experimental unit, as the treatments were applied into the jar, whereas treatment was included as the fixed effect and the denominator degrees of freedom was adjusted with the SATTHERTWAITE command of SAS. All analyses were performed in each individual sampling day (days 1 or 60) and reported as such. Moreover, the microbiological data (LAB, yeast, and mold) were log-transformed prior to the statistical evaluation. For the analyses conducted herein, all data were reported as least square means and treatment comparison adjusted for TUKEY. Significance was set at *p* ≤ 0.05, whereas tendencies were denoted if 0.05 < *p* ≤ 0.10.

## 5. Conclusions

Delaying the final ensiling process worsens fermentation indicators, increases dry matter (DM) losses, and reduces both aerobic stability and the hygienic quality of the silage. This highlights the importance of prompt ensiling to maintain silage quality. The use of inoculant additives, such as *Lentilactobacillus buchneri* and *Lactococcus lactis*, can be encouraged to minimize forage heating caused by delayed ensiling, reduce DM losses during fermentation, improve aerobic stability, and inhibit the growth of yeasts and molds in treated silages. The results of this study support the recommendation to minimize the duration of aerobic exposure of fresh forage during silo filling and to use LAB-based inoculants. This finding is valuable for farmers and researchers seeking to optimize ensiling practices for maize forage. However, further studies are warranted to compare the effects of different types and compositions of LAB-based additives on the characteristics of ensiled maize forage.

## Figures and Tables

**Table 1:** Maize forage temperature (°C) and contamination by yeast and mold before ensiling into mini-silo. Ambient temperature was 20 °C.

Item	T1C	T2C	T4I	T5I	SE	*p*-Value
Temperature after 24 h interruption	36.10 ^a^	33.18 ^b^	27.94 ^c^	25.08 ^d^	0.354	<0.01
Hours for the temperature to increase 3 °C versus ambient	6.0 ^c^	9.6 ^bc^	12.0 ^b^	18.0 ^a^	1.697	<0.01
Yeast, log	6.90 ^a^	6.67 ^a^	5.74 ^b^	5.66 ^b^	0.163	<0.01
Molds, log	5.94 ^a^	5.75 ^a^	4.42 ^b^	4.11 ^b^	0.177	<0.01
LAB, log	4.95 ^bc^	5.59 ^b^	6.92 ^a^	7.04 ^a^	0.356	<0.01

T1C—non-inoculated with ensiling pause for 24 h uncovered; T2C—non-inoculated with ensiling pause for 24 h covered-up; T4I—inoculated with ensiling pause for 24 h uncovered; T5I—inoculated with ensiling pause for 24 h covered-up; LAB—lactic acid bacteria (LAB, yeasts and mold, expressed as log_10_ cfu g^−1^); letters ^a,b,c,d^ in rows describe significant differences between treatments at *p* < 0.01.

**Table 2:** Nutrient composition of maize before ensiling into mini-silo under the conditions promptly ensiled or ensiled with interruption for 24 h with or without inoculant treatment (data given in g kg^−1^ DM unless stated otherwise).

Item	T0C	T1C	T2C	T3I	T4I	T5I	SE	*p*-Value
DM, g kg^−1^	365.8	366.1	366.2	366.9	366.6	366.6	3.856	1.000
CP	102.7	102.9	101.3	100.3	103.6	102.0	2.292	0.744
CF	198.4	197.1	196.4	197.7	197.5	195.2	3.651	0.966
WSC	76.6	74.1	76.0	77.8	76.5	77.5	4.963	0.983
Starch	328.4	326.1	324.1	327.4	329.4	326.4	8.435	0.992
ME, MJ	9.97	9.97	9.97	10.02	9.99	10.05	0.087	0.880
pH	5.74	5.72	5.72	5.74	5.69	5.68	0.032	0.331

T0C—non-inoculated promptly ensiled; T1C—non-inoculated with ensiling pause for 24 h uncovered; T2C—non-inoculated with ensiling pause for 24 h covered-up; T3I—inoculated and promptly ensiled; T4I—inoculated with ensiling pause for 24 h uncovered; T5I—inoculated with ensiling pause for 24 h covered-up; DM—dry matter; CP—crude protein; CF—crude fiber; WSC—water-soluble carbohydrates; ME—metabolizable energy.

**Table 3:** Nutrient composition and microbial characteristics of maize silage ensiled into mini-silo under the treatments promptly ensiled or ensiled with interruption for 24 h with or without inoculant treatment and fermented for 60 days (data given in g kg^−1^ DMc unless stated otherwise).

Item	T0C	T1C	T2C	T3I	T4I	T5I	SE	*p*-Value
DMc, g kg^−1^	348.8 ^bc^	345.8 ^c^	347.4 ^c^	356.2 ^a^	354.5 ^ab^	355.2 ^a^	3.085	<0.01
CP	92.7 ^ab^	88.4 ^c^	89.9 ^bc^	93.8 ^a^	90.6 ^abc^	91.6 ^abc^	1.147	0.01
WSC	13.6	11.9	11.5	13.8	16.2	12.7	2.189	0.335
Starch	331.1 ^abc^	326.6 ^c^	328.4 ^bc^	344.4 ^a^	339.4 ^abc^	336.6 ^abc^	4.694	<0.01
DM loss	61.63 ^a^	69.61 ^a^	64.54 ^a^	40.04 ^b^	44.39 ^b^	42.54 ^b^	2.863	<0.01
ME, MJ	9.56	9.50	9.52	9.62	9.56	9.60	0.044	0.687
Yeast, log_10_ cfu g^−1^	2.38 ^b^	3.30 ^a^	3.10 ^a^	1.40 ^c^	2.07 ^b^	1.99 ^b^	0.143	<0.01
Mold, log_10_ cfu g^−1^	2.23 ^b^	3.18 ^a^	3.03 ^a^	1.00 ^c^	1.28 ^c^	1.16 ^c^	0.096	<0.01
LAB, log_10_ cfu g^−1^	6.53 ^b^	6.51 ^b^	6.67 ^b^	7.92 ^a^	7.56 ^c^	7.78 ^a^	0.173	<0.01

T0C—non-inoculated promptly ensiled; T1C—non-inoculated with ensiling pause for 24 h uncovered; T2C—non-inoculated with ensiling pause for 24 h covered-up; T3I—inoculated and promptly ensiled; T4I—inoculated with ensiling pause for 24 h uncovered; T5I—inoculated with ensiling pause for 24 h covered-up; DMc—dry matter corrected for the loss of volatiles; CP—crude protein; WSC—water soluble carbohydrates; ME—metabolizable energy; LAB—lactic acid bacteria; letters ^a,b,c^ in rows describe significant differences between treatments at *p* < 0.01.

**Table 4:** Fermentation characteristics of maize silage ensiled into mini-silo under the treatments promptly ensiled or ensiled with interruption for 24 h with or without inoculant treatment and fermented for 60 days (data given in g kg^−1^ DMc unless stated otherwise).

Item	T0C	T1C	T2C	T3I	T4I	T5I	SE	*p*-Value
LA	46.59 ^bc^	38.36 ^d^	39.59 ^cd^	60.54 ^a^	49.78 ^b^	51.6 ^b^	2.320	<0.01
AA	16.6 ^c^	18.38 ^c^	17.42 ^c^	20.63 ^b^	24.70 ^a^	22.60 ^b^	0.642	<0.01
BA	0.42 ^ac^	1.12 ^a^	0.53 ^ac^	0.01 ^b^	0.07 ^b^	0.05 ^b^	0.280	<0.05
AL	26.70 ^c^	34.32 ^a^	31.07 ^b^	25.80 ^c^	24.49 ^c^	25.30 ^c^	0.815	<0.01
ETH	26.09 ^b^	30.87 ^a^	27.50 ^b^	15.52 ^c^	17.93 ^c^	17.60 ^c^	0.952	<0.01
Pp	1.93 ^d^	2.35 ^d^	2.11 ^d^	8.89 ^a^	4.37 ^c^	6.84 ^b^	0.209	<0.01
Ammonia-N, g kg^−1^ total N	52.68 ^b^	63.01 ^a^	55.37 ^ab^	38.03 ^c^	40.35 ^bc^	38.56 ^c^	3.208	<0.01
pH	3.88 ^bc^	3.98 ^a^	3.90 ^b^	3.80 ^d^	3.82 ^cd^	3.80 ^d^	0.020	<0.01

T0C—non-inoculated promptly ensiled, T1C—non-inoculated with ensiling pause for 24 h uncovered; T2C—non-inoculated with ensiling pause for 24 h covered-up; T3I—inoculated and promptly ensiled; T4I—inoculated with ensiling pause for 24 h uncovered; T5I—inoculated with ensiling pause for 24 h covered-up; LA—lactic acid, AA—acetic acid, BA—butyric acid, AL—alcohols; ETH—ethanol; Pp—1,2-propanediol; letters ^a,b,c,d^ in rows describe significant differences between treatments at *p* < 0.01 (or *p* < 0.05).

**Table 5:** Chemical composition of maize silage promptly ensiled or ensiled with a 24 h interruption, with or without inoculant treatment, at day 7 of aerobic exposure (data given in g kg^−1^ DMc unless stated otherwise).

Item	T0C	T1C	T2C	T3I	T4I	T5I	SE	*p*-Value
DMc, g kg^−1^	326.7 ^bc^	320.4 ^c^	327.1 ^bc^	346.6 ^a^	337.6 ^ab^	341.2 ^ab^	4.709	<0.01
CP	91.5	87.6	88.0	93.1	92.0	91.7	2.085	0.065
WSC	5.6	5.5	4.7	5.8	6.8	5.1	1.076	0.520
Starch	320.2 ^bcd^	310.1 ^d^	314.7 ^cd^	338.1 ^a^	333.0 ^ab^	329.7 ^abc^	5.269	<0.01
DM loss	148.93 ^a^	153.22 ^a^	133.58 ^a^	62.94 ^c^	89.09 ^b^	78.92 ^b^	10.575	<0.01
ME, MJ	9.30 ^b^	9.28 ^b^	9.32 ^b^	9.54 ^a^	9.56 ^a^	9.56 ^a^	0.034	<0.01

T0C—non-inoculated promptly ensiled; T1C—non-inoculated with ensiling pause for 24 h uncovered; T2C—non-inoculated with ensiling pause for 24 h covered-up; T3I—inoculated and promptly ensiled; T4I—inoculated with ensiling pause for 24 h uncovered; T5I—inoculated with ensiling pause for 24 h covered-up; DMc—dry matter corrected for the loss of volatiles; CP—crude protein; WSC—water soluble carbohydrates; ME—metabolizable energy; letters ^a,b,c,d^ in rows describe significant differences between treatments at *p* < 0.01.

**Table 6:** Fermentation characteristics of maize (silage promptly ensiled or ensiled with interruption for 24 h with or without inoculant treatment) at day 7 of the aerobic exposure (data given in g kg^−1^ DMc unless stated otherwise).

Item	T0C	T1C	T2C	T3I	T4I	T5I	SE	*p*-Value
LA	10.18 ^c^	6.44 ^c^	9.48 ^c^	42.19 ^a^	24.52 ^b^	32.12 ^b^	3.118	<0.01
AA	6.94 ^b^	6.36 ^b^	9.70 ^b^	17.36 ^a^	19.56 ^a^	20.64 ^a^	1.321	<0.01
BA	0.86 ^a^	0.55 ^ab^	1.00 ^a^	0.24 ^b^	0.36 ^b^	0.28 ^b^	0.154	<0.01
AL	6.20 ^b^	7.16 ^b^	10.76 ^a^	10.62 ^a^	9.14 ^ab^	11.54 ^a^	1.046	<0.01
ETH	4.26 ^bc^	4.94 ^b^	8.30 ^a^	2.64 ^c^	3.02 ^bc^	5.22 ^b^	0.736	<0.01
Pp	0.94 ^c^	1.41 ^c^	1.28 ^c^	7.20 ^a^	5.42 ^b^	5.56 ^b^	0.496	<0.01
Ammonia-N, g kg^−1^ total N	29.68 ^bc^	32.30 ^ab^	37.55 ^a^	27.58 ^bc^	25.74 ^c^	28.40 ^bc^	1.726	<0.01
pH	5.22 ^a^	5.36 ^a^	5.28 ^a^	3.90 ^c^	4.14 ^b^	4.10 ^b^	0.052	<0.01

T0C—non-inoculated promptly ensiled, T1C—non-inoculated with ensiling pause for 24 h uncovered; T2C—non-inoculated with ensiling pause for 24 h covered-up; T3I—inoculated and promptly ensiled; T4I—inoculated with ensiling pause for 24 h uncovered; T5I—inoculated with ensiling pause for 24 h covered-up; LA—lactic acid, AA—acetic acid, BA—butyric acid, AL—alcohols; ETH—ethanol; Pp—propanediol 1,2; letters ^a,b,c^ in rows describe significant differences between treatments at *p* < 0.01.

**Table 7:** Parameters of aerobic stability and microbiological characteristics of maize silage after aerobic exposure.

Item	T0C	T1C	T2C	T3I	T4I	T5I	SE	*p*-Value
AS, hours	144.0 ^c^	146.4 ^c^	171.6 ^c^	394.8 ^a^	312.0 ^b^	363.6 ^ab^	26.020	<0.01
HT, °C	27.72 ^a^	28.14 ^a^	27.34 ^a^	23.12 ^c^	25.12 ^b^	23.44 ^bc^	0.634	<0.01
	Microbial count at day 7 of aerobic exposure							
Yeast	8.21 ^a^	9.00 ^a^	8.22 ^a^	2.64 ^b^	2.88 ^b^	2.78 ^b^	0.357	<0.01
Mold	6.47 ^a^	6.18 ^a^	6.42 ^a^	1.95 ^b^	2.11 ^b^	2.03 ^b^	0.306	<0.01
	Microbial count at the end of 17 days aerobic exposure							
Yeast	9.02 ^a^	9.11 ^a^	9.03 ^a^	4.94 ^b^	5.09 ^b^	4.87 ^b^	0.453	<0.01
Mold	9.22 ^a^	9.38 ^a^	9.30 ^a^	3.10 ^b^	5.04 ^b^	3.85 ^b^	0.295	<0.01

T0C—non-inoculated promptly ensiled, T1C—non-inoculated with ensiling pause for 24 h uncovered; T2C—non-inoculated with ensiling pause for 24 h covered-up; T3I—inoculated and promptly ensiled; T4I—inoculated with ensiling pause for 24 h uncovered; T5I—inoculated with ensiling pause for 24 h covered-up; AS—aerobic stability; HT—highest temperature; letters ^a,b,c^ in rows describe significant differences between treatments at *p* < 0.01.

## Data Availability

The data presented in this study are available on request from the corresponding author.

## References

[1] Muck R.E., Nadeau E.M.G., McAllister T.A., Contreras-Govea F.E., Santos M.C., Kung L. (2018). Silage review: Recent advances and future uses of silage additives. J. Dairy Sci..

[2] Brüning D., Gerlach K., Weiß K., Südekum K.-H. (2018). Effect of compaction, delayed sealing and aerobic exposure on forage choice and short-term intake of maize silage by goats. Grass Forage Sci..

[3] Michel P.H.F., Gonçalves L.C., Rodrigues J.A.S., Keller K.M., Raposo V.S., Lima E.M., Santos F.P.C., Jayme D.G. (2017). Re-ensiling and inoculant application with *Lactobacillus plantarum* and *Propionibacterium acidipropionici* on sorghum silages. Grass Forage Sci..

[4] Wilkinson J.M., Muck R.E. (2019). Ensiling in 2050: Some challenges and opportunities. Grass Forage Sci..

[5] Weiss K., Kroschewski B., Auerbach H. (2022). The influence of delayed sealing and repeated air ingress during the storage of maize silage on fermentation patterns, yeast development and aerobic stability. Fermentation.

[6] dos Anjos G.V.S., Gonçalves L.C., Rodrigues J.A.S., Keller K.M., Coelho M.M., Michel P.H.F., Ottoni D., Jayme D.G. (2018). Effect of re-ensiling on the quality of sorghum silage. J. Dairy Sci..

[7] Mickan F.J., Martin M.D., Piltz J.W., Kaiser A.G., Piltz J.V., Burns H.M., Griffiths N.W. (2004). Silage storage. Successful Silage.

[8] Cai Y., Du Z., Yamasaki S., Nguluve D., Tinga B., Macome F., Oya T. (2020). Influence of microbial additive on microbial populations, ensiling characteristics, and spoilage loss of delayed sealing silage of Napier grass. Asian-Aust. J. Anim. Sci..

[9] Puntillo M., Gaggiotti M., Oteiza J.M., Binetti A., Massera A., Vinderola G. (2020). Potential of Lactic Acid Bacteria Isolated From Different Forages as Silage Inoculants for Improving Fermentation Quality and Aerobic Stability. Front. Microbiol..

[10] Drouin P., Tremblay J., Renaud J., Apper E. (2021). Microbiota succession during aerobic stability of maize silage inoculated with *Lentilactobacillus buchneri* NCIMB 40788 and *Lentilactobacillus hilgardii* CNCM-I-4785. MicrobiologyOpen.

[11] Saarisalo E., Jalava T., Skyttä E., Haikara A., Jaakkola S. (2006). Effect of lactic acid bacteria inoculants, formic acid, potassium sorbate and sodium benzoate on fermentation quality and aerobic stability of wilted grass. Agric. Food Sci..

[12] Borreani G., Tabacco E., Schmidt R.J., Holmes B.J., Muck R.E. (2018). Silage Review: Factors Affecting Dry Matter and Quality Losses in Silages. J. Dairy Sci..

[13] Weiss K., Kroschewski B., Auerbach H. (2016). Effects of air exposure, temperature and additives on fermentation characteristics, yeast count, aerobic stability and volatile organic compounds in corn silage. J. Dairy Sci..

[14] Pauly T., Sundberg M., Spörndly R., Udén P., Eriksson T., Rustas B.-O., Müller C.E., Spörndly R., Pauly T., Emanuelson M. (2013). Effects of delayed sealing during silo filling—Experiments with lab-scale silos. Proceedings of the 4th Nordic Feed Science Conference.

[15] Kim S.C., Adesogan A.T. (2006). Influence of Ensiling Temperature, Simulated Rainfall, and Delayed Sealing on Fermentation Characteristics and Aerobic Stability of Corn Silage. J. Dairy Sci..

[16] Krizsan S.J., Randby A.T. (2007). The effect of fermentation quality on the voluntary intake of grass silage by growing cattle fed silage as the sole feed. J. Anim. Sci..

[17] de Melo N.N., Carvalho-Estrada P.d.A., Tavares Q.G., Pereira L.d.M., Delai Vigne G.L., Camargo Rezende D.M.L., Schmidt P. (2023). The Effects of Short-Time Delayed Sealing on Fermentation, Aerobic Stability and Chemical Composition on Maize Silages. Agronomy.

[18] McDonald P., Henderson A.R., Heron S.J.E. (1991). The Biochemistry of Silage.

[19] Du Z., Risu N., Gentu G., Jia Y., Cai Y. (2020). Dynamic Changes and Characterization of the Protein and Carbohydrate Fractions of Native Grass Grown in Inner Mongolia during Ensiling and the Aerobic Stage. Asian-Aust. J. Anim. Sci..

[20] Hao W., Tian P., Zheng M., Wang H., Xu C. (2020). Characteristics of Proteolytic Microorganisms and Their Effects on Proteolysis in Total Mixed Ration Silages of Soybean Curd Residue. Asian-Aust. J. Anim. Sci..

[21] Hristov A.N., Bannink A., Crompton L.A., Huhtanen P., Kreuzer M., McGee M., Nozière P., Reynolds C.K., Bayat A.R., Yáñez-Ruiz D.R. (2019). Invited Review: Nitrogen in Ruminant Nutrition: A Review of Measurement Techniques. J. Dairy Sci..

[22] Auerbach H., Nadeau E. (2020). Effects of additive type on fermentation and aerobic stability and its interaction with air exposure on silage nutritive value. Agronomy.

[23] Kung L., Shaver R.D., Grant R.J., Schmidt R.J. (2018). Silage review: Interpretation of chemical, microbial, and organoleptic components of silages. J. Dairy Sci..

[24] Taylor C.C., Ranjit N.J., Mills J.A., Neylon J.M., Kung L. (2002). The effect of treating whole-plant barley with *Lactobacillus buchneri* 40788 on silage fermentation, aerobic stability, and nutritive value for dairy cows. J. Dairy Sci..

[25] Driehuis F., Oude Elferink S.J.W.H., Van Wikselaar P.G. (2001). Fermentation characteristics and aerobic stability of grass silage inoculated with Lactobacillus buchneri, with or without homofermentative lactic acid bacteria. Grass Forage Sci..

[26] Mills J.A., Kung L. (2002). The effect of delayed ensiling and application of a propionic acid-based additive on the fermentation of barley silage. J. Dairy Sci..

[27] Pahlow G., Muck R.E., Driehuis F., Oude Elferink S.J.W.H., Spoelstra S.F., Buxton D.R., Muck R.E., Harrison J.H. (2003). Microbiology of ensiling. Silage Science and Technology.

[28] Paradhipta D.H.V., Lee S.S., Kang B., Joo Y.H., Lee H.J., Lee Y., Kim J., Kim S.C. (2020). Dual-purpose inoculants and their effects on corn silage. Microorganisms.

[29] Kung L., Broderick G.A., Adesogan A.T., Bocher L.W., Bolsen K.K., Contreras-Govea F.E., Harrison J.H., Muck R.E. (2009). Potential factors that may limit the effectiveness of silage additives. Proceedings of the 15th International Silage Conference.

[30] Rooke J.A., Hatfield R.D., Buxton D.R., Muck R.E., Harrison J.H. (2003). Biochemistry of ensiling. Silage Science and Technology.

[31] Nutcher K., Salacci R., Kuber C.P., Kuber R., Uriarte M.E., Bolsen K.K., Daniel J.L.P., Morais G., Junges D., Nussio L.G. (2015). Effects of Sealing Time Post-Filling and Sealing Material on Fermentation, Nutritional Quality, and Organic Matter Loss of Whole-Plant Maize Ensiled in a Drive-over Pile. Proceedings of the XVII International Silage Conference.

[32] Alhaag H., Yuan X., Mala A., Bai J., Shao T. (2019). Fermentation characteristics of *Lactobacillus plantarum* and *Pediococcus* species isolated from sweet sorghum silage and their application as silage inoculants. Appl. Sci..

[33] Comino L., Tabacco E., Righi F., Revello-Chion A., Quarantelli A., Borreani G. (2014). Effects of an inoculant containing a Lactobacillus buchneri that produces ferulate-esterase on fermentation products, aerobic stability, and fiber digestibility of maize silage harvested at different stages of maturity. Anim. Feed Sci. Technol..

[34] Uriarte-Archundia M.E., Bolsen K.K., Brent B.E. (2002). A study of the chemical and microbial changes in whole-plant corn silage during fermentation and storage: Effects of packing density and sealing technique. Kans. Agric. Exp. Stn. Rep..

[35] Kung L., Ranjit N.K. (2001). The effect of Lactobacillus buchneri and other additives on the fermentation and aerobic stability of barley silage. J. Dairy Sci..

[36] Tabacco E., Piano S., Revello-Chion A., Boreanni G. (2011). Effect of *Lactobacillus buchneri* LN4637 and *Lactobacillus buchneri* LN40177 on the aerobic stability, fermentation products, and microbial populations of corn silage under farm conditions. J. Dairy Sci..

[37] (1998). Microbiology of Food and Animal Feeding Stuffs—Horizontal Method for the Enumeration of Mesophilic Lactic Acid Bacteria—Colony-Count Technique at 30 Degrees C.

[38] Jatkauskas J., Vrotniakiene V., Eisner I., Witt K.L., do Amaral R.C. (2024). Comparison of the Chemical andMicrobial Composition and Aerobic Stability of High-Moisture Barley Grain Ensiled with Either Chemical or Viable Lactic Acid Bacteria Application. Fermentation.

[39] Weissbach F.E., Strubelt C. (2008). Correcting the dry matter content of maize silages as a substrate for biogas production. Landtech. Net63.

[40] Pallauf J., SNP (Committee for Requirements Standards of the Society for Nutritional Physiology) (1998). Formulas for estimating the content of metabolizable energy in feed from permanent grassland and whole maize plants. (GfE (Ausschuss für Bedarfsnormen der Gesellschaft für Ernährungsphysiologie). Formeln zur Schätzung des Gehaltes an Umsetzbarer Energie in Futtermitteln aus Aufwüchsen des Dauergrünlandes und Mais-Ganzpflanzen) original. Proceedings of the Society Nutrition Physiology.

